# The Loss-of-Function Mutation *aldA67* Leads to Enhanced α-L-Rhamnosidase Production by *Aspergillus nidulans*

**DOI:** 10.3390/jof8111181

**Published:** 2022-11-09

**Authors:** Margarita Orejas, Andrew P. MacCabe

**Affiliations:** Instituto de Agroquímica y Tecnología de Alimentos (IATA), Consejo Superior de Investigaciones Científicas (CSIC), c/Catedrático Agustín Escardino Benlloch 7, 46980 Paterna, Valencia, Spain

**Keywords:** enzyme production, filamentous fungi, aldehyde dehydrogenase mutant *aldA67*, L-rhamnose catabolism, LRA pathway engineering, plant cell wall degrading enzymes (PCWDEs), RhaR transcriptional activator

## Abstract

In *Aspergillus nidulans* L-rhamnose is catabolised to pyruvate and L-lactaldehyde, and the latter ultimately to L-lactate, via the non-phosphorylated pathway (LRA) encoded by the genes *lraA*-*D*, and *aldA* that encodes a broad substrate range aldehyde dehydrogenase (ALDH) that also functions in ethanol utilisation. LRA pathway expression requires both the pathway-specific transcriptional activator RhaR (*rhaR* is expressed constitutively) and the presence of L-rhamnose. The deletion of *lraA* severely impairs growth when L-rhamnose is the sole source of carbon and in addition it abolishes the induction of genes that respond to L-rhamnose/RhaR, indicating that an intermediate of the LRA pathway is the physiological inducer likely required to activate RhaR. The loss-of-function mutation *aldA67* also has a severe negative impact on growth on L-rhamnose but, in contrast to the deletion of *lraA*, the expression levels of L-rhamnose/RhaR-responsive genes under inducing conditions are substantially up-regulated and the production of α-L-rhamnosidase activity is greatly increased compared to the *aldA*^+^ control. These findings are consistent with accumulation of the physiological inducer as a consequence of the loss of ALDH activity. Our observations suggest that *aldA* loss-of-function mutants could be biotechnologically relevant candidates for the over-production of α-L-rhamnosidase activity or the expression of heterologous genes driven by RhaR-responsive promoters.

## 1. Introduction

Plant biomass resulting from the photosynthetic fixation of atmospheric carbon dioxide is the natural renewable source of terrestrial carbon. The lignocellulosic fraction of this biomass is composed of plant cell wall (PCW) materials, of which cellulose, hemicelluloses and pectins are the polysaccharidic components and account for >70% of its total dry mass [1]. Hemicelluloses and pectins, unlike cellulose, are structurally heterogeneous, and this heterogeneity extends not only between plant species but also to the different structural elements of individual plants [2].

In the natural environment, fungi and other microorganisms use PCW polysaccharides as sources of carbon and energy. These are accessed through the activity of microbial extracellular enzymes that deconstruct the PCW polymers releasing sugars suitable for cellular uptake and assimilation via appropriate catabolic pathways. L-rhamnose is a deoxy-hexose (6-deoxy-L-mannopyranose) that is present in the pectic polysaccharides rhamnogalacturonan (RG) I and RGII, as well as in hemicelluloses and the seaweed polymer ulvan [3 and references therein]. This sugar can be used as a nutrient by fungi and other microorganisms and can also provoke changes in gene expression. The release of L-rhamnose from PCW polymeric substrates is catalysed by extracellular enzymes, including α-L-rhamnosidases, the production of which is induced in its presence. α-L-Rhamnosidases (E.C. 3.2.1.40; GH78) cleave terminal α-L-rhamnose residues from a wide range of natural products and have important applications in the food, pharmaceutical, cosmetics and fine-chemical industries (e.g., fruit juice de-bittering, wine-aroma improvement, enhancement of the bioavailability or the pharmacological/biological activity of natural glycosides, and the extraction of L-rhamnose for use as a feedstock for chemical synthesis or as an anti-ageing agent), as well as playing a role in vivo in counteracting plant defences against pathogens [3,4,5,6,7,8,9,10]. These enzymes occur widely in nature and are found in animal tissues, plants, yeasts, fungi and bacteria (BRENDA Enzyme Database (brenda-enzymes.org); [11] and references therein).

**Figure 1:**
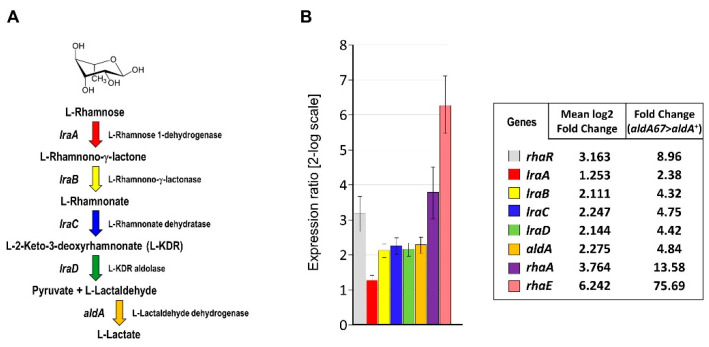
Enhanced expression of the RHA/RhaR genes in the *aldA* loss-of-function mutant *aldA67* under inducing conditions: (**A**) biochemical route (LRA) of L-rhamnose catabolism in *A. nidulans*; (**B**) relative transcript abundance in the *aldA67* mutant (AR463) compared to the control strain AR462 (*aldA*^+^) after transfer of mycelia to inducing conditions (1% L-rhamnose), as determined by RT-qPCR.

Of the three pathways known for the catabolism of L-rhamnose (Types I, II and III) [12], our recent work has established the exclusive operation of the Type II non-phosphorylated pathway (LRA) in the filamentous fungus *Aspergillus nidulans*. This pathway is encoded by five genes: *lraA* (AN4186; L-rhamnose-1-dehydrogenase), *lraB* (AN3740; L-rhamnono-γ-lactonase), *lraC* (AN5672; L-rhamnonate dehydratase), *lraD* (AN9425; L-2-keto-3-deoxyrhamnonate (L-KDR) aldolase of the HpcH family; PF03328/COG3836), and *aldA* (AN0554; aldehyde dehydrogenase—ALDH). The products of the *lra* genes yield L-pyruvate and L-lactaldehyde, and subsequently ALDH catalyses the conversion of L-lactaldehyde to L-lactate (Figure 1A—see Section 3.1). Deletion of *lraA* resulted in the inability to grow on L-rhamnose and loss of the production of α-L-rhamnosidases, thus demonstrating not only the indispensability of this pathway for L-rhamnose utilization but also establishing that the true physiological inducer of the genes involved in L-rhamnose metabolism is a catabolite derived from this sugar and not the sugar itself [13,14]. Induction of the expression of the catabolic genes and the genes encoding the two characterised α-L-rhamnosidases (RhaA and RhaE) is mediated by the pathway-specific transcriptional activator RhaR in the presence of L-rhamnose [13,14,15,16,17,18]. The observation that *rhaR* is expressed in the absence of L-rhamnose [18] suggests that the physiological inducer activates extant RhaR, a situation not dissimilar to that seen for xylanase gene induction in which the presence of xylose is required for transcriptional activation mediated by XlnR [19]. Recent studies in *Aspergillus niger* have suggested L-KDR to be the physiological inducer of the genes involved in L-rhamnose utilisation [20].

Characterisation of the genes encoding a catabolic pathway and knowledge of their regulation can provide a conceptual framework on which to base design strategies directed to modulating the productivity of fungal cell factories. By way of example, an *Aspergillus niger* strain deleted for the *lraA* and *lraC* homologues was shown to facilitate the extraction of L-rhamnose from both refined (naringin and rhamnogalacturonan) and non-refined (grape fruit peel) substrates since the mutant was able to release L-rhamnose from these materials but was unable to utilise the liberated sugar, thus resulting in its accumulation [21]. This demonstrated the feasibility of a biotechnological alternative to the labour-intensive, expensive and environmentally harmful methods traditionally used for the extraction and chemical hydrolysis of plant raw materials in order to obtain L-rhamnose [22].

Our studies in *A. nidulans* concerning the metabolic fate of the L-lactaldehyde generated from the reaction catalysed by LraD provided strong evidence for the involvement of an L-lactaldehyde dehydrogenase (LADH) activity that is encoded by the well-studied ethanol/*alc*-regulon gene *aldA* (AN0554; aldehyde dehydrogenase—ALDH, [23]). RhaR-dependent induction of *aldA* was observed when L-rhamnose was present as the sole source of carbon [14], and growth of an *aldA67* loss-of-function mutant [24,25] on rhamnose was seen to be very severely impaired compared to that of the wild type, whereas growth of both strains on glucose was identical. In the present study, we have examined the molecular background of the crucial role played by *aldA* in L-rhamnose metabolism and its potential biotechnological relevance. Whilst there are many examples of genetic manipulations directed to improving extracellular enzyme production by filamentous fungi, such as the generation of multicopy transformants, promoter modification or swapping, transcription factor mutagenesis, heterologous expression, etc., our findings constitute a novel biotechnological approach for achieving significant enzyme overproduction (i.e., rhamnosidase activity or activities driven by RhaR-responsive promoters).

## 2. Materials and Methods

### 2.1. Strains and Growth Conditions

*A. nidulans* strains AR462 (*aldA^+^*, *pabaA1*, *yA2*), AR463 (*aldA67*, *pabaA1*, *yA2*), AR271 (*ΔnkuA*::*argB*, *argB2*, *riboB2*::*Af_riboB*, *pyroA4*) and AR247 (*ΔnkuA*::*argB*, *argB2*, *ΔlraA*::*Af_riboB*, *riboB2*, *pyroA4*) were used in this study.

For transfer experiments, mycelial biomass was generated from an inoculum of 5 × 10^6^ conidia/mL in minimal medium (MM; [26]) containing 0.5% yeast extract, supplemented with 5 mM urea (nitrogen source), 0.1% (*w/v*) D-fructose (sole carbon source) and 2 µg/mL PABA from concentrated stocks. After 18 h growth at 37 °C with orbital shaking at 180 rpm, ~1.5 g of mycelium was harvested, washed with MM lacking a carbon source, drained and transferred to new medium (see Section 3). Induction medium (15 mL) was prepared by substituting D-fructose by 1% (*w*/*v*) L-rhamnose in the original growth medium. For solid media, 1.5% agar (OXOID) was added.

### 2.2. RNA Isolation and RT-qPCR

The procedures used for isolating total RNA and undertaking RT-qPCR are described in detail in [13]. The relative quantification of reference-gene-normalised target genes was determined using the Relative Expression Software Tool (Multiple Condition Solver REST-MCS v2) [27]. Oligonucleotides used for RT-qPCR are listed in Table S1.

### 2.3. α-L-Rhamnosidase Assays

Extracellular α-L-rhamnosidase activity was measured in an assay based on the hydrolysis of *p*-nitrophenyl α-L-rhamnopyranoside (*p*NPR). Reactions were carried out in 96-well plates, and the release of *p*-nitrophenol was measured at 400 nm using a Clariostar spectrophotometer. Assays were performed for 15 min at 50 °C in final volumes of 250 μL using 1.4 mM substrate in McIlvaine buffer pH 4.0 [28]. Reactions were stopped by adding an equal volume (250 µL) of 0.25 M sodium carbonate.

To qualitatively assess the production of α-L-rhamnosidase activity of *A. nidulans* colonies in vivo in the absence of L-rhamnose, MM plates supplemented with 10 mM sodium nitrate (nitrogen source), 2 µg/mL PABA, 0.5 µg/mL pyridoxine and 1% (*w*/*v*) lactose (sole carbon source) were spread with 100 µL of 10 mM 4-methylumbelliferyl α-L-rhamnopyranoside (MUR) in McIlvaine buffer pH 4.0. After incubation of inoculated plates for 65 h at 25 °C, the hydrolysis of MUR was visualised by illumination with UV light.

## 3. Results

### 3.1. The aldA67 Loss-of-Function Mutation Affects the Expression of Genes Involved in L-Rhamnose Utilisation

The enzyme encoded by the *A. nidulans aldA* gene is known to be involved in regulating the expression of genes of the *alc* regulon by modulating the intracellular concentration of their physiological co-inducer, acetaldehyde [25]. The latter is derived from ethanol via the activity of the alcohol dehydrogenase ADHI and then converted by ALDH to acetate, which is subsequently assimilated into central metabolism as acetyl-CoA. We recently reported genetic evidence for a novel and essential role for the *aldA* gene product in L-rhamnose catabolism [14]—the conversion of L-lactaldehyde to lactate—and in this regard it is noteworthy that earlier biochemical studies of ALDH showed it to be an enzyme of broad rather than narrow aldehyde substrate specificity [24,29]. We also demonstrated previously that the physiological inducer of rhamnose utilisation is an intermediate of the LRA pathway [13]. Given that blockage of a step in a metabolic pathway (e.g., as a consequence of a mutant allele) can provoke the accumulation of upstream intermediates leading to loss of (or modulation of) flux through the pathway, we have investigated the possible influence of *aldA* on the expression of genes involved in L-rhamnose catabolism. To this end, the relative transcript abundances of eight genes (*rhaR*, *lraA-D*, *aldA*, *rhaA* and *rhaE*) were assessed by RT-qPCR in mycelia of both the *aldA67* mutant (AR463) and its *aldA*^+^ isogenic control (AR462) after the transfer of each from non-inducing/non-repressing medium (0.1% fructose) to a medium containing 1% L-rhamnose as the sole carbon source (inducing conditions). The *aldA67* mutant was found to have elevated expression of all the genes tested, including that of the mutant allele itself (Figure 1B). Of particular interest for potential biotechnological applications are the very considerable increases observed in the transcript abundances of the α-L-rhamnosidase genes (~14- and ~76-fold for *rhaA* and *rhaE* respectively), and to a lesser but nonetheless noteworthy extent that of the transcriptional activator RhaR (~9-fold). The latter could be indicative of intracellular accumulation of the inducing catabolite (the co-inducer) and autoregulation of *rhaR*. The greater transcript abundance of the *aldA67* mutant under inducing conditions (Figure 1B) is suggestive of its potential as a fungal cell factory for enhanced production of the α-L-rhamnosidases RhaA and RhaE.

**Figure 2:**
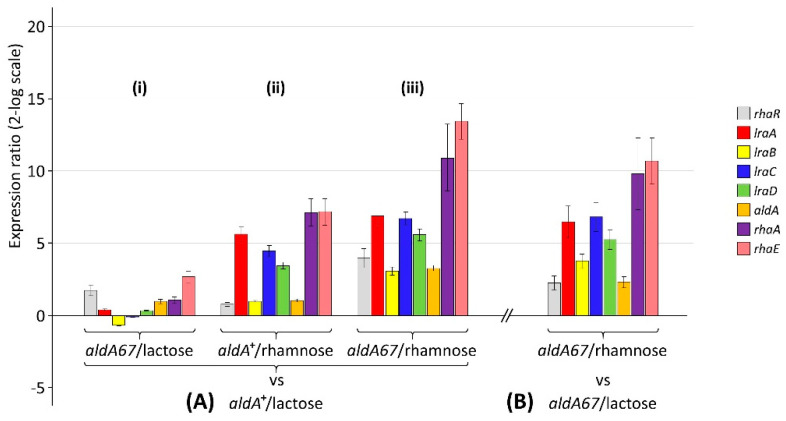
Effect of the *aldA67* allele on the expression of genes involved in L-rhamnose utilisation under inducing and non-inducing conditions: (**A**) relative transcript abundance of *aldA*^+^ and *aldA67* mycelia under inducing and non-inducing conditions vs. the wild-type strain in non-inducing medium (panels (**i**–**iii**) are described in the text); (**B**) relative transcript abundance of *aldA67* mycelia under inducing conditions vs. the *aldA67* strain in non-inducing medium.

In order to obtain a more detailed picture of the effects of the *aldA67* mutation on the genes involved in L-rhamnose utilisation in the absence of the inducing carbon source, RT-qPCR was also carried out on mycelia transferred to medium containing 1% lactose as the sole (non-inducing) source of carbon. Comparing transfers of *aldA*^+^ and *aldA67* mycelia to lactose (Figure 2A(i)), there are relatively minor fluctuations (~2-fold or less) in transcript accumulation in most of the genes analysed except for the regulatory gene *rhaR* and the structural gene *rhaE,* where abundances were greater in the *aldA67* mutant by ~3- and ~6-fold, respectively. In the comparison of the *aldA*^+^ strain transferred to L-rhamnose on the one hand and lactose on the other (Figure 2A(ii)), the patterns of increased transcript accumulation under inducing vs. non-inducing conditions are very similar to those reported previously for *lraA-C*, *rhaA* and *rhaE* in the alternative ‘wild-type’ strain AR5 (*biA1*) [13]. Compared to the *aldA*^+^ strain transferred to lactose, the greatest relative induction of expression is seen in the *aldA67* mutant after transfer to L-rhamnose (Figure 2A(iii))—most notably for *rhaR* and the genes encoding the α-L-rhamnosidases. Figure 2B shows the result of transfer of the *aldA67* mutant to rhamnose vs. lactose for comparison.

### 3.2. The aldA67 Mutation Results in Greater α-L-Rhamnosidase Production

**Figure 3:**
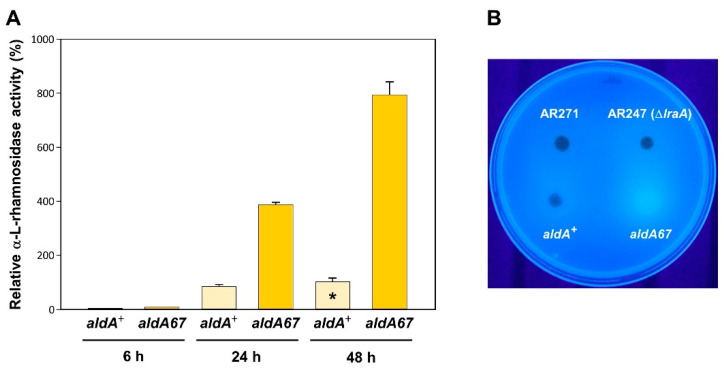
Enhanced extracellular α-L-rhamnosidase activity in the *aldA* loss-of-function mutant *aldA67*: (**A**) time course of extracellular α-L-rhamnosidase activity produced by control AR462 (*yA2*, *pabaA1*) and mutant AR463 (*aldA67*, *pabaA1*, *yA2*) mycelia in liquid culture after transfer to inducing conditions (1% L-rhamnose) assayed using the artificial substrate 4-nitrophenol α-L-rhamnopyranoside (*p*NPR). Activity (means + standard deviation) is expressed as a percentage relative to that of the control strain at 48 h * (100%). Measurements were made in duplicate on three biological replicates. (**B**) qualitative assessment of extracellular α-L-rhamnosidase activity on non-inducing solid medium (1% lactose) as revealed by hydrolysis of 4-methylumbelliferyl α-L-rhamnopyranoside (MUR); 2 µL drops of conidial suspensions at a titre of 10^7^ conidia/mL were spotted.

To evaluate the *aldA67* loss-of-function mutant as a potential over-producer of α-L-rhamnosidase activity, the kinetics of its extracellular rhamnosidase production in liquid (shake flask) culture were compared to those of the *aldA*^+^ control strain 6, 24 and 48 h after transfer of mycelial mass from 0.1% fructose medium to inducing conditions (medium containing 1% L-rhamnose). As can be seen in Figure 3A, α-L-rhamnosidase activity produced by the mutant strain after 24 and 48 h was dramatically increased compared to the control (*aldA*^+^ at 48 h), reaching levels ~4- and ~8-fold higher, respectively.

A plate test was also carried out to visually assess the extracellular production of α-L-rhamnosidase activity on non-inducing (1% lactose) solid (agar) medium supplemented with the fluorogenic artificial substrate 4-methylumbelliferyl α-L-rhamnopyranoside (MUR): 2 µL drops (≡2 × 10^4^ conidia) of conidial suspensions of the *aldA67* mutant, its *aldA*^+^ control, an *lraA*-deleted strain (AR247—does not produce rhamnosidase activity; [13]) and the latter’s *lraA*^+^ isogenic control strain (AR271) were spotted onto the agar surface. After incubation (see Materials and Methods), the plate was exposed to UV illumination to visualise MUR hydrolysis (Figure 3B). As expected, neither the strain deleted for *lraA* nor the *lraA^+^* control exhibited fluorescence halos and hence did not produce extracellular enzymatic activity. Whilst the same was also true for the *aldA*^+^ isogenic control, the halo around the *aldA67* mutant revealed the production of extracellular α-L-rhamnosidase activity despite the sole availability of a non-inducing carbon source. This observation is concordant with the increased abundance of *rhaE* transcripts seen in the *aldA67* mutant in non-inducing (1% lactose) shake flask culture (Figure 2A panel (i)) and suggestive of the phenomenon of ‘pseudo-constitutive’ expression as a consequence of the accumulation of a co-inducing compound under non-inducing conditions.

## 4. Discussion

Using a genetic approach, we recently demonstrated a novel and crucial role for the *alc* regulon gene *aldA* in the LRA pathway for L-rhamnose catabolism in which its gene product (ALDH) catalyses the conversion of L-lactaldehyde to lactate [14]. Expression of the *alc* genes in the presence of ethanol is mediated by the transcriptional activator AlcR, and acetaldehyde is its co-inducer. The level of acetaldehyde in the cell is governed by ALDH that irreversibly catalyses its conversion to acetate, and *aldA* loss-of-function mutations result in pseudo-constitutive expression of the *alc* regulon, i.e., transcriptional activation under non-inducing conditions due to accumulation of the co-inducer [25]. In the current work, we have demonstrated the very considerable capacity of the *aldA67* loss-of-function mutant for α-L-rhamnosidase over-production associated with enhanced *lra*/*rha* gene transcription under inducing (L-rhamnose) conditions. In addition, and reminiscent of the behaviour of the *alc* regulon, the production of this activity and the transcriptional up-regulation of certain other genes involved in L-rhamnose catabolism under non-inducing conditions has also been revealed. Given the role of *aldA* in the LRA pathway, the *aldA67* mutant allele can be expected to result in the accumulation of L-lactaldehyde, without excluding the possibility of accumulation of other intermediates.

The L-lactaldehyde formed by the LRA pathway (Figure 1A) derives from the action of the final L-rhamnose-specific catalytic activity of this route (L-KDR aldolase—encoded by *lraD*/AN9425) on L-2-keto-3-deoxyrhamnonate (L-KDR). The constitutively expressed *A. nidulans rhaR* gene (AN5673) encodes the transcriptional activator (RhaR) of genes involved in L-rhamnose utilisation [18], and the failure of the deletion of the *lraD* homologue in *A. niger* to impede the induction of the expression of genes under the control of RhaR has been taken to indicate L-KDR to be its corresponding co-inducer [20]. The accumulation of L-lactaldehyde in the *aldA67* mutant could in turn lead to accumulation of L-KDR due either to feedback inhibition of cleavage of the aldol or, given that the reaction catalysed by LraD is reversible, the LraD-mediated aldol condensation of L-lactaldehyde with pyruvate, resulting in the production of L-2-keto-3-deoxysugar acid intermediates including L-KDR [30]. The interaction of the accumulating co-inducer with RhaR in the *aldA67* mutant under non-inducing conditions could thus result in pseudo-constitutive expression of genes that are regulated by RhaR. Indeed, the pattern of pseudo-constitutive gene expression observed (Figure 2A panel (i)) is congruent with the pattern of RhaR-mediated gene induction on L-rhamnose [13].

In conclusion, the *A. nidulans aldA67* mutant shows increased levels of transcription of RhaR target genes when cultured under either non-inducing or inducing conditions, and especially so in the latter where it achieves very considerable α-L-rhamnosidase over-production compared to the *aldA*^+^ control. Given the utility of α-L-rhamnosidases in diverse food and other industrial applications, and extrapolating this finding to filamentous fungal production strains, the selection for (or generation of) loss-of-function mutations in *aldA* could yield strain variants providing enhanced enzyme yields. Strains carrying non-engineered *aldA* mutations (e.g., *aldA67*: a classical mutation that truncates AldA at position Trp131 of the 497 translation product; [24,25]) could be employed for the production of enzymatic activities, the uses of which may only be acceptable from non-GMO sources. By contrast, in the case of GMOs, a gene encoding a specifically desired enzyme activity or protein/peptide could be placed under the control of a strongly responding RhaR-target promoter (e.g., *rhaE*_p_) and introduced into an *aldA* loss-of-function mutant for over-expression.

## Data Availability

The data presented in this study are available on request from the corresponding authors.

## Supplementary Materials

The following supporting information can be downloaded at https://www.mdpi.com/article/10.3390/jof8111181/s1, Table S1: Oligonucleotides used in this study.
